# Low-temperature-processed efficient semi-transparent planar perovskite solar cells for bifacial and tandem applications

**DOI:** 10.1038/ncomms9932

**Published:** 2015-11-18

**Authors:** Fan Fu, Thomas Feurer, Timo Jäger, Enrico Avancini, Benjamin Bissig, Songhak Yoon, Stephan Buecheler, Ayodhya N. Tiwari

**Affiliations:** 1 Laboratory for Thin Films and Photovoltaics, Empa—Swiss Federal Laboratories for Materials Science and Technology, Ueberlandstrasse 129, 8600 Duebendorf, Switzerland; 2 Laboratory for High Performance Ceramics, Empa—Swiss Federal Laboratories for Materials Science and Technology, Ueberlandstrasse 129, 8600 Duebendorf, Switzerland

## Abstract

Semi-transparent perovskite solar cells are highly attractive for a wide range of applications, such as bifacial and tandem solar cells; however, the power conversion efficiency of semi-transparent devices still lags behind due to missing suitable transparent rear electrode or deposition process. Here we report a low-temperature process for efficient semi-transparent planar perovskite solar cells. A hybrid thermal evaporation–spin coating technique is developed to allow the introduction of PCBM in regular device configuration, which facilitates the growth of high-quality absorber, resulting in hysteresis-free devices. We employ high-mobility hydrogenated indium oxide as transparent rear electrode by room-temperature radio-frequency magnetron sputtering, yielding a semi-transparent solar cell with steady-state efficiency of 14.2% along with 72% average transmittance in the near-infrared region. With such semi-transparent devices, we show a substantial power enhancement when operating as bifacial solar cell, and in combination with low-bandgap copper indium gallium diselenide we further demonstrate 20.5% efficiency in four-terminal tandem configuration.

## Abstract (web summary)

Perovskite solar cells already exhibit large efficiencies above 20%. Here, the authors use a low temperature sputtering process to fabricate semi-transparent perovskite solar cells, demonstrating bifacial operation and a 4-terminal tandem with CIGS solar cells surpassing single junction cells.

## Graphical abstract

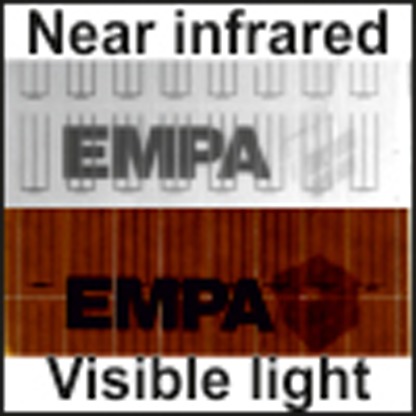

The emerging perovskite solar cells have received increasing attention due to the high efficiency, easy processing and potentially low cost^1^,^2^. Although the power conversion efficiency of perovskite solar cells have soared up to 20.1% (ref. 3), there is still ample room for further efficiency improvement through novel concepts^4^,^5^,^6^. An effective approach to increase the efficiency and lower the production cost is to make semi-transparent solar cells that can convert solar energy into electricity from both front and rear side of the device^7^. The bifacial cell concept has been implemented in various kinds of solar cell technologies^8^,^9^,^10^,^11^,^12^, and up to 50% output power enhancement has been demonstrated in Si wafer bifacial modules by collecting the albedo radiation from surroundings^13^. In addition, the semi-transparent perovskite solar cells hold great promise for applications in tandem solar cells^14^, photon energy upconversion^15^, building-integrated photovoltaics^16^, wearable electronics^17^, powering sensors and electronic gadgets in homes.

There are still many challenges hindering the realization of high-performance semi-transparent perovskite solar cells. The state-of-the-art perovskite solar cells^3^,^18^,^19^,^20^,^21^,^22^ employ high-temperature-processed (∼500 °C) TiO_2_ (mesoporous and compact) as electron transporting layer (ETL), which is incompatible with monolithic tandem or flexible solar cells on plastics. Thus, it is desirable to develop a planar structure that allows low-temperature processing. It is well known that perovskite solar cells, particularly in planar configuration with TiO_2_ as ETL, suffer from pronounced hysteresis in the current density–voltage (*J*–*V*) curve, which often results in overestimated device performance^23^. Therefore, it is imperative to eliminate the hysteresis, which is not only important to ensure a stable performance but also to unambiguously evaluate the real device efficiency. [6,6]-phenyl-C_61_-butyric acid methyl ester (PCBM) has been proven to assist in eliminating the *J*–*V* hysteresis in inverted device configuration^24^. Several efforts have been made in positioning PCBM in regular device structure; however, the hysteresis phenomenon still remains^25^,^26^. For bifacial and tandem solar cell applications, it is crucial to replace the commonly used metal contacts in perovskite cells by highly transparent conducting electrodes, which allow sunlight incident from front and rear side of the device and to transmit the photons with energy below the bandgap of the perovskite. Previously, thin layers of Au (ref. 27), carbon nanotubes^28^ and PEDOT:PSS^29^ have been explored as transparent contact for perovskite solar cells, while the efficiencies are generally below 10% and these contacts featured strong absorption in near infrared (NIR). Efficient semi-transparent devices have been realized with graphene rear electrode; however, all the devices show strong hysteresis in current density–voltage curves^30^,^31^. Recently, sputtered transparent conductive oxides (TCO), including indium tin oxide (ITO)^32^, ZnO:Al^33^ and indium zinc oxide (IZO)^34^, have been reported with highest efficiency of up to 12.1% for a semi-transparent cell and 19.5% in perovskite–CIGS (copper indium gallium diselenide) four-terminal configuration^33^. Bailie *et al*.^35^ developed a 12.7% efficiency semi-transparent perovskite solar cell employing Ag nanowires, and demonstrated 18.6 and 17% tandem efficiency by combining with CIGS and low-quality silicon bottom cell, respectively. Despite the high NIR transparency and low sheet resistance of Ag nanowires, the complicated deposition method leads to large efficiency variation and poor reproducibility, and the fast diffusion of Ag into Spiro-OMeTAD and perovskite results in degradation of device stability and performance^36^. Therefore, the implementation of a suitable transparent electrode material and deposition process is the key and challenge to high-efficiency semi-transparent perovskite solar cells. An ideal transparent electrode for perovskite solar cells should meet the following requirements: superior transparency in visible and NIR region, low sheet resistance, robust adhesion, good chemical stability and compatibility with adjoining layers. As the perovskite and Spiro-OMeTAD are temperature and solvent sensitive^35^, a low-temperature and dry process would be favoured for the deposition of transparent electrodes. In this regard, the room-temperature sputtering deposited TCO would be an ideal candidate.

Here we report a low-temperature (≤50 °C)-processed hysteresis-free high-efficiency semi-transparent planar perovskite solar cell. The hysteresis is eliminated through a PCBM interlayer and high-quality perovskite absorber enabled by the hybrid thermal evaporation–spin coating method. High-mobility hydrogenated indium oxide (In_2_O_3_:H) is employed as transparent rear electrode through a room-temperature radio-frequency magnetron sputtering, which enables a semi-transparent device with steady-state efficiency of 14.2% and 72% average transmittance between 800 and 1,150 nm. Such a device when operated as a bifacial cell yields an additional 13.5% power output improvement, and 20.5% efficiency is demonstrated using perovskite top cell and CIGS bottom cell in four-terminal tandem configuration.

## Results

### Low-temperature-processed planar perovskite solar cells

**Figure 1:**
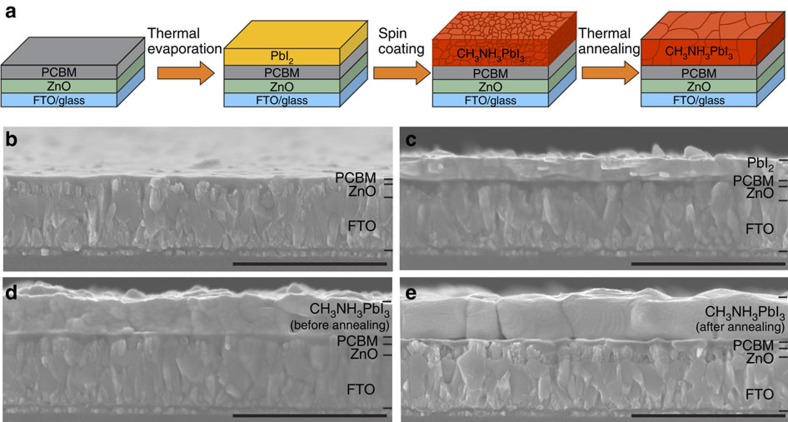
Hybrid thermal evaporation–spin coating preparation method and corresponding microstructure. (**a**) Deposition of perovskite layer through a combined thermal evaporation of PbI_2_ and spin coating of CH_3_NH_3_I solution in isopropanol followed by 2 h annealing at 50 °C on hotplate. (**b**–**e**) The cross-sectional SEM images of each layer during perovskite preparation. Scale bars, 1 μm in **b**–**e**.

Figure 1 shows a schematic drawing and cross-sectional scanning electron microscopy (SEM) images of the perovskite absorber deposited by hybrid thermal evaporation–spin coating method. The high-temperature-processed TiO_2_ is replaced by ∼100 nm compact ZnO layer deposited through radio-frequency magnetron sputtering at room temperature, followed by spin coating of ∼50 nm PCBM. The room-temperature-processed ETL is attractive for low-cost, lightweight and flexible plastic substrates, and compatible with monolithic tandem fabrication. As illustrated in Fig. 1a, the perovskite layer is formed through a hybrid evaporation solution approach, where a compact PbI_2_ layer is initially deposited by thermal evaporation^37^. This method allows a uniform and compact PbI_2_ deposited over large area. Then CH_3_NH_3_I in isopropanol solution is added by spin coating, followed by thermal annealing at 50 °C for 2 h on hotplate. Afterwards, ∼200 nm Spiro-OMeTAD is spin coated on top of the perovskite. Finally, the cell is finished by evaporating 60 nm Au for opaque reference solar cells. For semi-transparent solar cells, thermally evaporated MoO_3_ and room-temperature-sputtered In_2_O_3_:H are employed as rear contact. Thus, the highest processing temperature during the opaque and semi-transparent device fabrication is not higher than 50 °C. More detailed fabrication process information can be found in the Methods section.

For planar perovskite solar cells, a uniform and compact perovskite layer is essential for achieving high efficiency^38^. As ZnO conformally covers the rough fluorine-doped tin oxide (FTO) surface, the deposition of PCBM smoothens the surface where the perovskite is grown (Fig. 1b). Consequently, the thermally evaporated PbI_2_ exhibits a compact and uniform layer over large area (Fig. 1c). Figure 1d,e presents the cross-sectional SEM images of perovskite layer before and after annealing, showing that the perovskite layer retained the desired compact morphology from parent PbI_2_. With thermal annealing, the grain size increases considerably from several tens of nanometres to values comparable to the film thickness (Fig. 1d,e), which might be beneficial to the transport and recombination properties of the absorber.

The herein introduced perovskite preparation method offers several merits. In previously reported two-step methods where PbI_2_ is spin coated from solution, it is quite challenging to control the morphology of the PbI_2_ film deposited from solution on planar substrates^39^. The low solubility of PbI_2_ in the commonly used solvent *N*,*N*-dimethylformide^40^ could limit the thickness variation and the choice of the underlying substrates where PbI_2_ is deposited. We overcome these challenges by using a simple thermal evaporation method, which can deposit a compact and uniform PbI_2_ layer with precisely controlled thickness over large area. The room-temperature vapour deposition has no constraints on the underlying substrates, which opens up numerous possibilities for exploring other solvent-sensitive carrier-selective contacts and novel cell structure. For instance, this process allows the introduction of PCBM below the perovskite layer, which is crucial to eliminate the *J*–*V* hysteresis^24^, but difficult to realize in solution process as the commonly used polar solvents, such as *N*,*N*-dimethylformide, dimethylsulfoxide or γ-butyrolactone, for perovskite formation are detrimental to thin layers of PCBM.

### Planar perovskite solar cells without *J*–*V* hysteresis

**Figure 2:**
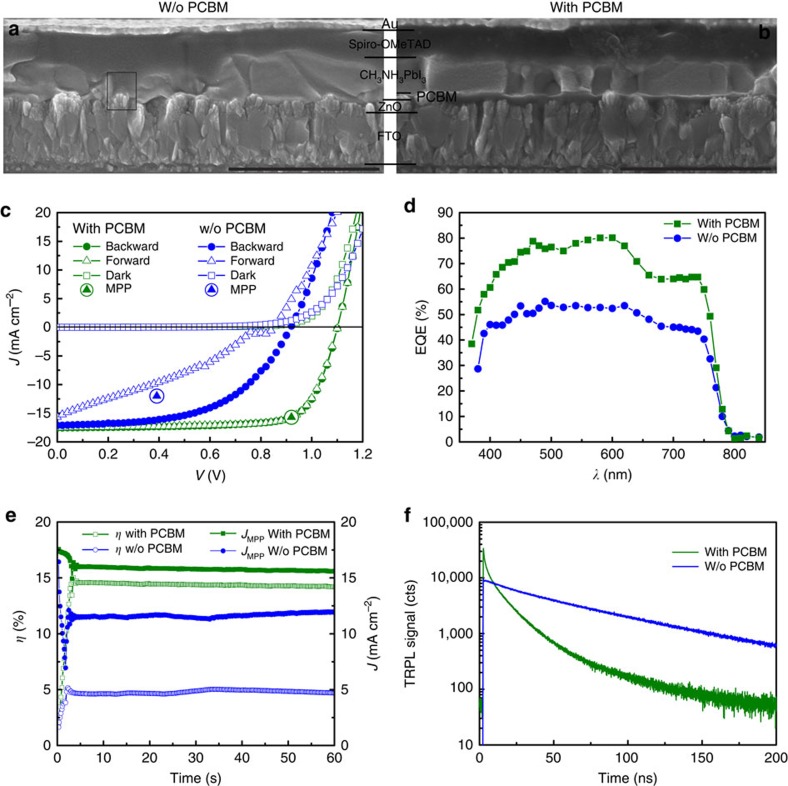
Microstructure, time-resolved photoluminescence and photovoltaic performance of planar perovskite solar cells. (**a**,**b**) The cross-sectional SEM images of devices without (**a**) and with (**b**) PCBM, respectively. The rectangular area in **a** indicates shunting path. (**c**–**e**) The current density–voltage (*J*–*V*) curves (**c**), EQE spectra (**d**) and steady-state efficiency at maximum power point (**e**) of the planar perovskite solar cells. All the devices are measured under simulated AM1.5G irradiation with 1,000 W m^−2^ intensity. The *J*–*V* measurements are performed in both forward (−0.1 to 1.2 V) and backward (1.2 to −0.1 V) direction at a scan rate and delay time of 0.3 V s^−1^ and 10 ms, respectively. The steady-state efficiency is evaluated by using a maximum power point tracker algorithm with constant AM1.5G illumination. (**f**) Time-resolved photoluminescence for samples with and without PCBM layer. Scale bars, 1 μm in **a** and **b**. W/o, without.

The hybrid thermal evaporation–spin coating method enables us to investigate the influences of PCBM on perovskite microstructure and device performance in regular planar configuration. Figure 2a,b presents the cross-sectional SEM images of the planar perovskite solar cell with and without PCBM layer, respectively. Other than PCBM, the devices are fabricated by an identical process with perovskite layer grown from 120 nm PbI_2_ (estimated by quartz microbalance) and 40 mg ml^−1^ CH_3_NH_3_I solution. It can be seen from the SEM images that the perovskite layer shows considerable surface roughness and thickness non-uniformity when grown directly on ZnO, which leads to low-resistance shunting paths and insufficient light absorption. A uniform and compact perovskite layer with large grain size is obtained when grown on PCBM. The microstructural difference in perovskite layers is mainly attributed to the morphological differences in PbI_2_ layers, as shown in Supplementary Fig. 1. If PbI_2_ is grown on ZnO directly, porous layers comprising numerous nanoplates are obtained^41^. This could form lots of grain boundaries and defects after the conversion into perovskite. It is important to note that high-efficiency devices produced by the here described process always contain residual PbI_2_ as shown in Supplementary Fig. 2. The existence of residual PbI_2_ is also observed in many high-efficiency devices reported in literature and several beneficial effects, for example, grain boundary passivation, hole-blocking effect and so on, have been proposed^42^,^43^.

Figure 2c shows the *J*–*V* characteristics of the corresponding planar perovskite solar cells under simulated AM1.5G irradiation. Owing to the hysteresis in *J*–*V* measurements^31^, it is crucial to report both forward (short circuit to forward bias) and backward (forward bias to short circuit) measurements along with the measurement conditions. The photovoltaic parameters are summarized in Supplementary Table 1. The device with PCBM layer shows an open circuit voltage (*V*_oc_) of 1.101 V, short circuit current density (*J*_sc_) of 17.6 mA cm^−2^ and fill factor (FF) of 74.9%, resulting in power conversion efficiency (*η*) of 14.5% in forward scan. In backward scan a *V*_oc_ of 1.103 V, *J*_sc_ of 17.5 mA cm^−2^, FF of 74.6% and *η* of 14.4% are obtained. The *J*–*V* curves from forward and backward scans coincide well, indicating no *J*–*V* hysteresis is observed in the devices. The *J*_sc_ is confirmed by external quantum efficiency (EQE) measurement (Fig. 2d) with an integrated *J*_sc_ of 17.4 mA cm^−2^ under AM1.5G spectrum. Furthermore, the efficiency measured at the maximum power point (MPP), that is, the steady-state output of the device, is shown in Fig. 2e. The cell exhibits a steady-state efficiency of 14.4% and current density of 15.7 mA cm^−2^ at MPP under continuous illumination. This is consistent with the *J*–*V* and EQE measurements. The device without PCBM layer generally shows a lower *V*_oc_, *J*_sc_ and FF with pronounced *J*–*V* hysteresis, and the steady-state efficiency lies between forward and backward efficiency. The integrated *J*_sc_ of 12.1 mA cm^−2^ is also lower than that derived from the *J*–*V* measurement.

Time-resolved photoluminescence decays are presented in Fig. 2f to probe the effect of PCBM on carrier dynamics in perovskite solar cells. In both cases the decay tails allow to estimate a minority carrier lifetime of ∼80 ns (ref. 44). Furthermore, it can be seen that the addition of PCBM leads to a nonlinear initial PL transient, which can be explained by charge-separating fields^45^. This indicates that the application of PCBM promotes a working junction without the need of initial biasing. Therefore, the elimination of *J*–*V* hysteresis and enhanced photovoltaic performance primarily stem from improved interface and junction formation and from the high-quality perovskite layer with enhanced crystallinity, uniform and compact morphology with large grain size.

### Semi-transparent planar perovskite solar cells

For semi-transparent cells, the opaque Au is substituted by In_2_O_3_:H transparent electrodes. In_2_O_3_:H is a high-mobility TCO material with superior visible and NIR transmittance^46^, and excellent thermal and chemical stability for solar cell applications were reported^47^. Deposition of In_2_O_3_:H by sputtering at room temperature without post-annealing results in layers with reasonably high mobility of 51.3 cm^2^ V^−1^ s^−1^ and sheet resistance of 25.7 Ω per square for 149 nm thick (determined from profilometer) amorphous layers. The effect of post-annealing treatment on the electrical properties (sheet resistance, electron mobility and electron density) and optical properties (transmission and absorption) of In_2_O_3_:H films on glass is presented in Supplementary Fig. 3. The amorphous nature of the as-sputtered In_2_O_3_:H is confirmed by X-ray diffraction (Supplementary Fig. 4). The radio-frequency sputtering method is a high-throughput and industrial scale method. However, high energetic ion bombardment during sputtering can damage the underlying layers. This is especially the case for organic layers such as Spiro-OMeTAD. Two different TCOs, that is, ZnO:Al and In_2_O_3_:H, are deposited by radio-frequency magnetron sputtering with different extent of ion bombardment and compared as transparent electrode.

**Figure 3:**
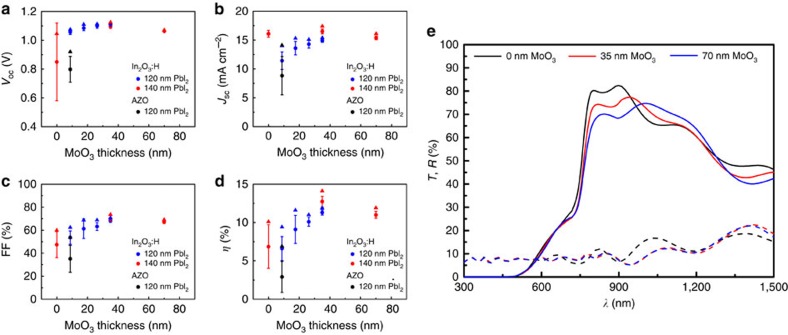
Photovoltaic and optical properties of semi-transparent planar perovskite solar cells with different MoO_3_ thickness. (**a**–**d**) The photovoltaic parameters of semi-transparent planar solar cells as a function of MoO_3_ thickness with In_2_O_3_:H as rear transparent electrode. The photovoltaic performances of device with ZnO:Al rear contact are also presented for comparison. Average values and s.d. are given from 5 to 9 cells (cell area: 0.3 cm^2^ for In_2_O_3_:H; and 0.15 cm^2^ for ZnO:Al). The triangle symbol represents the highest value for each batch. The *J*–*V* measurement conditions were the same as in Fig. 2. (**e**) The transmission (*T*) and reflection (*R*) of semi-transparent cells with various MoO_3_ thicknesses. The solid and dashed lines represent transmission and reflection, respectively. The perovskite is grown from 140 nm PbI_2_ precursor. The MoO_3_ thickness used here is determined from SEM images.

The degree of ion bombardment is more severe during the deposition of ZnO:Al than that in In_2_O_3_:H due to the different sputter geometries, lower total pressure and shorter target-to-substrate distance^48^, as illustrated in Supplementary Fig. 5. In both devices, 8.7 nm MoO_3_ is evaporated as buffer layer, and perovskite is grown from 120 nm-thick PbI_2_ precursor. As can be seen in Fig. 3a,d, the average *V*_oc_, *J*_sc_, FF and *η* of devices with ZnO:Al are much lower than that of the ones with In_2_O_3_:H, and the s.d. is also larger in case of ZnO:Al. Notably, a perovskite solar cell with 10.1% efficiency (Supplementary Fig. 6) is achieved by directly depositing In_2_O_3_:H on top of Spiro-OMeTAD. This is the highest value with direct deposition of TCO on top of the hole transporting layer (HTL) as back electrode without buffer layer, indicating that direct sputtered TCO might allow to achieve high-efficiency if the ion bombardment is suppressed during deposition.

In the current device architecture and deposition procedure, the use of MoO_3_ proves still to be beneficial. Figure 3 shows the photovoltaic and optical properties as a function of the MoO_3_ thickness as well as the schematic cell structure of semi-transparent planar perovskite solar cell. A general increase of all the photovoltaic parameters combined with a substantial increase in uniformity is observed with increasing MoO_3_ thickness up to 35 nm. Further, it is shown that the *J*_sc_ is less sensitive to MoO_3_ thickness variation if the PbI_2_ precursor thickness is increased to 140 nm while the FF and *V*_oc_ exhibit the same trend as devices grown from 120 nm PbI_2_ precursors. For MoO_3_ thicknesses above 35 nm no increase is observed anymore. The highest *V*_oc_ and FF in semi-transparent cells are comparable to opaque reference ones, indicating a reasonable trade-off between minimized sputtering damage and added series resistance with 35 nm MoO_3_ thickness. In addition to the photovoltaic performance, the MoO_3_ thickness also affects the transmission and reflection spectra as shown in Fig. 3e. The transmission shows a decreasing trend with increasing MoO_3_ thickness between 800 and 940 nm, while an opposite trend with thickness variation between 940 and 1,200 nm. The device without MoO_3_ shows a peak transmission of 82%, while device with 35 nm MoO_3_ shows the highest average transmission in 800–1,150 nm region. Therefore, an optimal 35 nm-thick MoO_3_ layer is deposit on Spiro-OMeTAD before In_2_O_3_:H deposition in terms of photovoltaic and optical properties in the following work.

**Figure 4:**
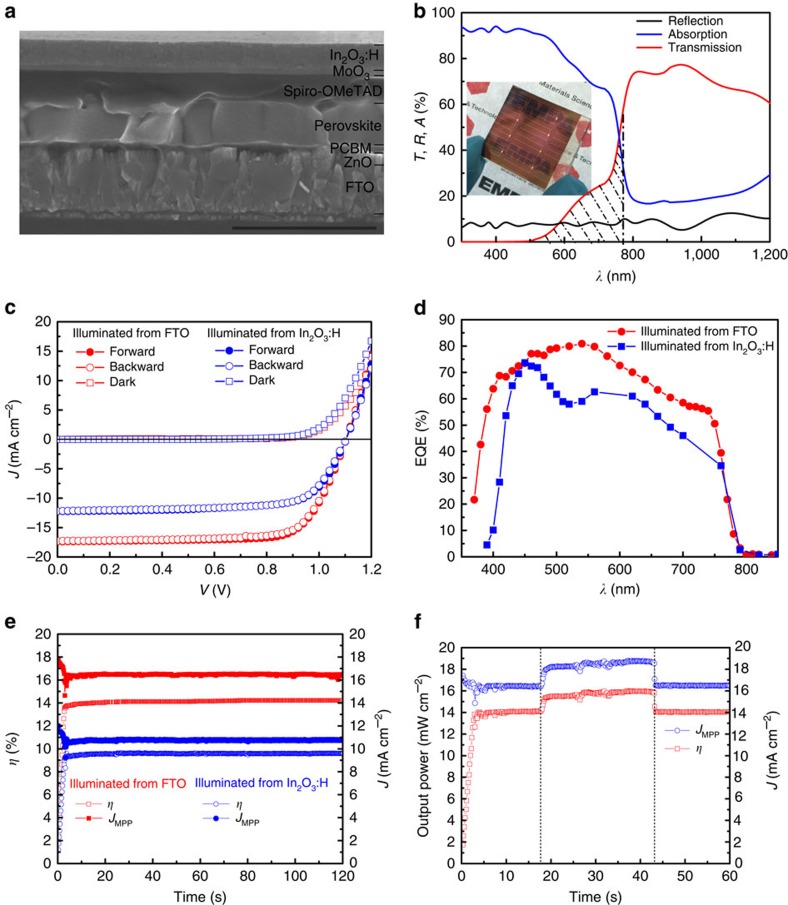
Microstructure and photovoltaic performance of the semi-transparent planar perovskite solar cell. (**a**) The cross-sectional SEM image of the complete device. Scale bar, 1μm. (**b**) The transmission (*T*), reflection (*R*) and absorption (*A*) of semi-transparent cell. A photograph of the semi-transparent device is also shown as inset. Current density–voltage (*J*–*V*) curve (**c**), external quantum efficiency (EQE) spectra (**d**) and steady-state efficiency at maximum power point (**e**) of the semi-transparent planar perovskite solar cell. (**f**) Bifacial application of the semi-transparent cell with white paper as reflective background. The first and second dashed lines indicate the insertion and removal of the commercial white paper as reflective background, respectively. The measurement conditions in the *J*–*V*, EQE and MPP are same as in Fig. 2.

Figure 4 summarizes the microstructure, transmission and photovoltaic performance of a semi-transparent planar perovskite solar cell produced as described above. The cross-sectional SEM image in Fig. 4a shows a cell structure of FTO/ZnO/PCBM/CH_3_NH_3_PbI_3_/Spiro-OMeTAD/MoO_3_/In_2_O_3_:H. An electron beam-evaporated Ni-Al grid is applied for better charge carrier collection in cells with area above 0.5 cm^2^. The cell area is defined by mechanical scribing down to the FTO layer. The perovskite grown from 140 nm PbI_2_ and 45 mg ml^−1^ CH_3_NH_3_I solution shows a flat and dense layer with grain size comparable to the film thickness. Figure 4b displays the transmission, reflection and absorption of the semi-transparent cell. The transmission through the whole device shows a peak of 77% at 940 nm and an average of 72% between 800 and 1,150 nm. The high sub-bandgap transmission is attributed to the low free-carrier absorption in high-mobility In_2_O_3_:H (ref. 48). In addition, the photograph of the semi-transparent device in the inset of Fig. 4b shows a decent transmission in visible region as the picture behind the device can be clearly recognized. The hatched area in Fig. 4b indicates optical losses due to insufficient absorption in the perovskite layer. This means there is still much room to increase the current in the semi-transparent cell by optimizing the (optical) thickness of perovskite layer.

Figure 4c,d presents the *J*–*V* curves and EQE spectra of the semi-transparent planar perovskite solar cell illuminated from front (FTO) and rear (In_2_O_3_:H) side. The forward and backward scans show negligible *J*–*V* hysteresis. The corresponding photovoltaic parameters are summarized in Supplementary Table 1. The semi-transparent device shows a *V*_oc_ of 1.104 V, *J*_sc_ of 17.4 mA cm^−2^, FF of 73.6% and *η* of 14.1% illuminated from FTO, and *V*_oc_ of 1.105 V, *J*_sc_ of 12.2 mA cm^−2^, FF of 70.6% and *η* of 9.5% illuminated from In_2_O_3_:H, as shown in Fig. 4c. The *V*_oc_ remains the same regardless of the illumination side, while higher *J*_sc_ and FF are observed when illuminated from FTO side. The EQE spectra are consistent with the *J*_sc_ discrepancy in *J*–*V* curves, where rear illumination shows a lower EQE, particularly below 430 nm. This distinct difference can be well explained by the strong parasitic absorption of Spiro-OMeTAD (Supplementary Fig. 7). Strong interference and the shading effect of metal grid also contribute to the lower current density when illuminated from rear side. The semi-transparent cell shows a high *V*_oc_ of 1.1 V, comparable to the reference cell. The FF is slightly reduced due to the lower conductivity of the In_2_O_3_:H/Ni-Al grid contact compared with Au. Generally, the current density in the semi-transparent cell is lower than that in the opaque cell when the perovskite layer is grown from same PbI_2_ thickness (120 nm) as shown in Supplementary Fig. 8. To verify the efficiency measured from *J*–*V*, the steady-state output at MPP is measured and presented in Fig. 4e. On illumination, the current density and efficiency increase within 3 s (ramp up of the MPP tracker) to 16.4 mA cm^−2^ and 14.2% for front illumination, and 10.8 mA cm^−2^ and 9.6% for rear illumination, respectively. The steady-state power conversion efficiency of 14.2% is the highest reported value for semi-transparent planar perovskite solar cell to date, which is particularly remarkable due to the simultaneously high transparency in NIR region. In addition, the semi-transparent cell with In_2_O_3_:H showed improved air stability compared with the opaque cell with Au in a preliminary stability experiment, as shown in Supplementary Fig. 9. In the following, two of many applications of high-efficiency semi-transparent perovskite solar cells are discussed in more detail.

### Bifacial planar perovskite solar cell

Bifacial solar cells are capable of converting sunlight incident from the front and the rear side of the cell into electricity, therefore the power output could be improved if highly reflective surrounding is present behind rear side of the cell. To illustrate how the power output gain works, we simply put a white paper from a commercial notebook as reflective surrounding under rear side of the cell during the MPP measurement. As shown in Fig. 4f, the power output quickly increased from original 14.1 to 16.0 mW cm^−2^ after adding the white paper behind the solar cell, while it dropped back to 14.1 mW cm^−2^ after removing the white paper. Using white paper as reflective surrounding gives 13.5% performance enhancement. By using a more reflective background and proper arrangement of solar modules in a system, the output power gain can be further improved up to 50% as demonstrated in silicon technology by Ceuvas *et al*.^13^.

### Four-terminal perovskite–CIGS tandem solar cell

A four-terminal mechanically stacked tandem device is demonstrated based on perovskite as top subcell and CIGS as bottom subcell, as schematically shown in Supplementary Fig. 10. The CIGS absorber layer is fabricated by a multistage co-evaporation process^49^. In a four-terminal tandem device, the top and bottom subcells are individually processed and mechanically stacked together. As the top cell and bottom subcell are electrically independent, two MPP trackers can be used to ensure optimum operation of each subcell at any condition. This also means that the efficiency of each subcell can be evaluated separately, and the sum of the two gives the efficiency of the device in tandem configuration. In this work, the performance of the bottom CIGS is directly measured with perovskite as a filter under standard illumination (Supplementary Fig. 10). The cell area of the bottom CIGS is defined by a laser-scribed shadow mask with aperture of 0.213 cm^2^ inserted between the top and bottom subcells.

**Figure 5:**
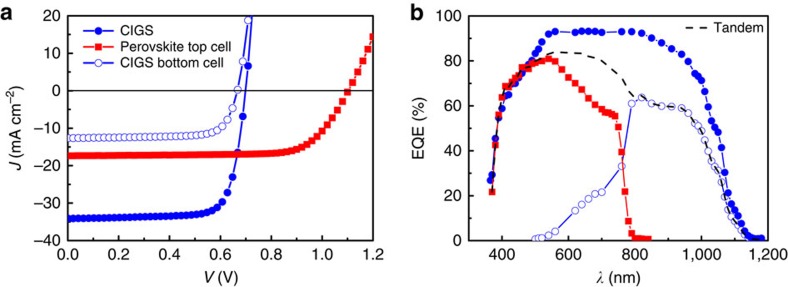
Photovoltaic performance of the four-terminal perovskite–CIGS tandem solar cell. (**a**,**b**) The current density–voltage (*J*–*V*) curves (**a**) and EQE spectra (**b**) of the four-terminal tandem device.

The *J*–*V* curves and EQE spectra of the top and bottom subcells are displayed in Fig. 5, and the photovoltaic parameters are summarized in Table 1. For comparison, the photovoltaic performance of the CIGS without perovskite filter is also presented. After adding the perovskite top cell as filter, the *J*_sc_ of the bottom CIGS decreased as expected due to reduced light intensity. The FF and *V*_oc_ are only marginally reduced, resulting in 6.3% efficiency for the CIGS bottom cell. Together with the 14.2% efficiency of the perovskite top, this result in 20.5% efficiency for the device in tandem configuration, which is a substantial improvement compared with each single-junction efficiency as well as to what was reported earlier for polycrystalline thin-film solar cells in tandem configuration^32^,^33^,^34^,^35^,^50^,^51^,^52^.

Currently, the transmission is still limited by the strong parasitic absorption in the front FTO electrode. The low-temperature process presented here facilitates the use of more transparent TCOs, such as In_2_O_3_:H or indium zinc oxide. The transmission and absorption spectra of In_2_O_3_:H, ZnO:Al and commercially available FTO and ITO are compared in Supplementary Fig. 11. The In_2_O_3_:H has the lowest absorption among all the TCOs investigated here between 300 and 2,000 nm. This implies there is much room in further lowering the parasitic absorption of semi-transparent perovskite solar cell in NIR region if front FTO is replaced by high-mobility In_2_O_3_:H. Since both the fabrication process for the CIGS solar cell and the for the perovskite cell are compatible to deposition on polymer films, lightweight and bendable polycrystalline tandem devices are in reach. Optimization of the bandgap of top and bottom cells, application of anti-reflection coating and optical coupling media can lead to even higher tandem efficiency in the near future as discussed in more detail by Kranz *et al*.^33^.

## Discussion

In conclusion, a low-temperature-processed high-efficiency semi-transparent planar perovskite solar cell without *J*–*V* hysteresis is developed by using sputtered hydrogenated indium oxide (In_2_O_3_:H) as transparent rear electrode. The high-temperature-processed (∼500 °C) TiO_2_ is replaced by room-temperature-sputtered ZnO, and the perovskite is prepared by a combined thermal evaporation of PbI_2_ and spin coating of CH_3_NH_3_I followed by 2-h annealing at 50 °C, thus making the maximum processing temperature as low as 50 °C. The hybrid thermal evaporation–spin coating method enables the introduction of PCBM in regular device configuration, which results in a high-quality perovskite layer and better electron collection, thus leads to a 14.5% hysteresis-free planar perovskite solar cell. High-mobility In_2_O_3_:H is employed as transparent rear electrode deposited by sputtering at room temperature, which enables a semi-transparent planar perovskite solar cell with high steady-state efficiency of 14.2% and simultaneous 72% average transmission between 800 and 1,150 nm. Finally, we show a 13.5% output power enhancement when the semi-transparent cell functioned as bifacial solar cell and demonstrate 20.5% efficiency device with perovskite top cell and CIGS bottom cell in four-terminal tandem configuration. This work has significant beneficial implications in lightweight tandem solar cells, bifacial solar cell, photon energy upconversion, photovoltaic-integrated buildings and wearable/portable electronics.

## Methods

### Perovskite solar cell fabrication

Perovskite solar cells were grown on FTO-coated glass (Pilkington, 15 Ω per square.) substrates. The 5 cm × 5 cm FTO/glasses were washed by hand followed by ultrasonic soap and water baths, and then cut into four quarters. Subsequently, a 100 nm compact ZnO layer was deposited at room temperature by radio-frequency magnetron sputtering on top of the FTO/glass at a deposition pressure of 6 × 10^−3^ mbar with Ar and O_2_ flow of 45 and 16 sccm, respectively. The deposition power density was 1.9 W cm^−2^, and the deposition time was 4.5 min. Then 30 μl of PCBM solution (20 mg ml^−1^ in chlorobenzene) was spin coated on top of ZnO at 2,000 r.p.m. for 40 s in the glovebox. The PbI_2_ film was thermally evaporated on rotating PCBM/ZnO/FTO/glass substrates at a deposition pressure of 2–5 × 10^−8^ mbar. Unless otherwise noted, the deposition rate is controlled within 1.2–1.6 Å s^−1^, monitored by a quartz crystal microbalance. The thicknesses of PbI_2_ precursors for opaque cells is 120 nm. For semi-transparent devices, 120 and 140 nm were used. After the PbI_2_ deposition, the samples were subsequently transferred into a N_2_-filled glovebox for further processing. The perovskite layer was formed by spin coating of CH_3_NH_3_I (Dyesol, 99.9%) in 2-propanol at a concentration of 40 and 45 mg ml^−1^ for 120 and 140 nm PbI_2_, respectively. The solution was first spread to cover the whole substrate, and the rotation (2,000 r.p.m. for 40 s) was started after 10 s. The as-prepared films were annealed at 50 °C for 2 h on a hotplate inside the glovebox. After annealing, the samples were cooled down to room temperature and 30 μl of a Spiro-OMeTAD solution (72.3 mg 2,2′,7,7′-tetrakis-(*N*,*N*′-di-*p*-methoxyphenylamine)-9,9′-spirobifluorene (Spiro-OMeTAD; Merck), 17.5 μl lithium-bis(trifluoromethanesulfonyl)imide (Li-TFSI, Sigma-Aldrich) solution (520 mg Li-TFSI in 1 ml acetonitrile, Sigma-Aldrich) and 28.8 μl 4-tertbutylpyridine (TBP, Sigma-Aldrich) all dissolved in 1,000 μl chlorobenzene (Sigma-Aldrich)) was spin coated on top of the perovskite at 2,000 r.p.m. for 40 s. The devices were finished by evaporating 60 nm Au through a metal mask under high vacuum (<5 × 10^−6^ mbar), defining the cell area of 0.15 cm^2^ for reference cells. For semi-transparent devices, a 35 nm thick MoO_3_ was deposited on top of Spiro-OMeTAD via thermal evaporation, which was covered by 170 nm of In_2_O_3_:H as transparent electrode. Ni/Al/Ni grids with 50 nm/2,000 nm/50 nm thickness were deposited by electron beam evaporation. Finally, the cell area (0.517 cm^2^) is defined by mechanical scribing. No anti-reflection coating is applied for the semi-transparent cells.

### Radio-frequency sputtering of hydrogenated indium oxide

Hydrogenated indium oxide (In_2_O_3_:H) layers were deposited in a high vacuum sputtering system (AJA Int.) by radio-frequency sputtering of ceramic In_2_O_3_ (99.99%) targets at an applied sputter power density of 3.0 W cm^−2^ without intentional heating of the substrate. The reactive atmosphere consisted of a gas mixture of Ar, O_2_ and H_2_O at a total pressure of 0.6 Pa. H_2_O vapour for H doping was injected via a needle valve with a partial pressure of ∼1 × 10^−4^ Pa.

### Radio-frequency sputtering of aluminium doped zinc oxide

Aluminium-doped zinc oxide (ZnO:Al) layers were deposited in a high-vacuum sputtering system by radio-frequency sputtering of ceramic ZnO (containing 2 wt% Al_2_O_3_) targets. The sputtering deposition consist of a 5-min deposition ramp up from 0.6 to 2.5 W cm^−2^ and followed by 5 × 3 min at 2.5 W cm^−2^ under 20 sccm Ar and 0.29 sccm Ar/O_2_ (3 mol% O_2_). There is a 30-min waiting time during each step to alleviate the temperature effect.

### X-ray diffraction measurements

X-ray diffraction patterns were obtained in Bragg–Brentano geometry by using a X'Pert PRO *θ*–2*θ* scan (Cu-K_α1_ radiation, *λ*=1.5406 Å) from 10 to 60° (2*θ*) with a step interval of 0.0167°.

### Scanning electron microscopy

The cross-sectional images of the samples were investigated with a Hitachi S-4800 using 5 kV voltage and 10 mA current. A thin layer of Pt was coated on top of the sample to avoid charging effect.

### Ultraviolet–visible spectroscopy

The transmission (T) and reflection (R) spectra were acquired using a ultraviolet–visible-NIR spectrophotometer (Shimadzu UV-3600) equipped with an integrating sphere. Absorption *A* is calculated using the following formula: *A*=1−*T*−*R.*

### Time-resolved photoluminescence

For time-resolved photoluminescence measurements, a 639 nm diode laser with pulse duration of 92 ps was used as excitation source. Pulse repetition rates were around 1 MHz and the number of photons per area and pulse was ∼1 × 10^13^ photons per cm^2^. The spectrally integrated luminescence decay was measured using a Picoquant PMA-C 192-M photomultiplier time and a Picoquant TimeHarp 260 digitizing system with 50 ps time channel width.

### Hall effect measurements

Hall effect measurements of In_2_O_3_:H films were performed with a HMS 3,000 Hall effect measurement system at room temperature in the dark using van der Pauw geometry. The electron density and electron mobility were calculated from Hall measurements.

### Solar cell performance characterization

The current density–voltage characteristics of perovskite solar cells were measured under standard simulated AM1.5G illumination using a Keithley 2,400 source meter. The illumination intensity was calibrated to 1,000 W m^−2^ using a certified single crystalline silicon solar cell. The *J*–*V* measurement is performed in both forward (form −0.1 V to 1.2 V) and backward (from 1.2 V to −0.1 V) directions separately without any pretreatment (for example, light soaking, holding at forward bias for certain time and so on). The scan rate and delay time are 0.3 V s^−1^ and 10 ms, respectively. The external quantum efficiency of the devices were measured with a lock-in amplifier. The probing beam was generated by a chopped white source (900 W, halogen lamp, 260 Hz) and a dual grating monochromator. The beam size was adjusted to ensure that the illumination area was fully inside the cell area. A certified single crystalline silicon solar cell was used as the reference cell. White light bias was applied during the measurement with ∼0.1 sun intensity. The steady-state efficiency as a function of time was recorded using a maximum power point tracker, which adjusts the applied voltage in order to reach the maximum power point (perturb and observe algorithm). The starting voltage is set to be 0.1 V.

## Additional information

**How to cite this article:** Fu, F. *et al*. Low-temperature-processed efficient semi-transparent planar perovskite solar cells for bifacial and tandem applications. *Nat. Commun.* 6:8932 doi: 10.1038/ncomms9932 (2015).

## Supplementary Material

Supplementary Information Supplementary Figures 1-11 and Supplementary Table 1.

## Figures and Tables

**Table 1:** Photovoltaic parameters of the four-terminal perovskite–CIGS tandem solar cells.

**Solar cells**	***V***_**OC**_ **(mV)**	***J***_**SC**_ **(mA cm**^**−2**^**)**	***Calculated J***_**SC**_ **(mA cm**^**−2**^**)**	**FF (%)**	*η* **(%)**	**Steady-state efficiency (%)**
Perovskite top subcell	1,104	17.4	16.7	73.6	14.1	14.2
CIGS cell (stand alone)	698.6	34.1	34.9	76.7	18.3	18.3
CIGS bottom subcell	667.4	12.7	12.4	74.9	6.3	6.3
Four-terminal tandem cell						20.5
