# Supplementary material for: Low-temperature-processed efficient semi-transparent planar perovskite solar cells for bifacial and tandem applications
Source: Nat Commun. 2015 Nov 18;6:8932. doi: 10.1038/ncomms9932 (PMC4696455; doi:10.1038/ncomms9932)
Supplement: Supplementary Information — Supplementary Figures 1-11 and Supplementary Table 1. [file ncomms9932-s1.pdf]

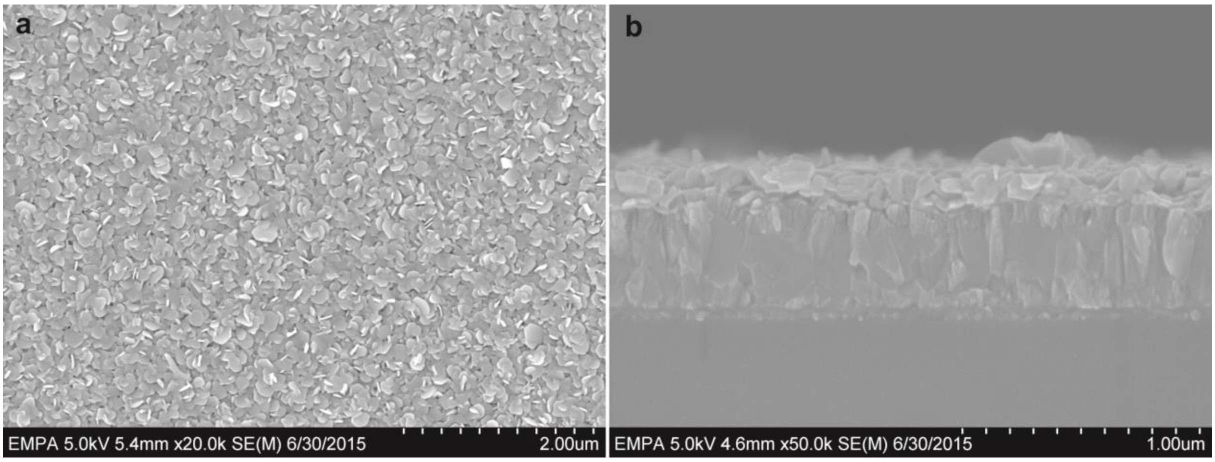

Supplementary Figure 1 | SEM iamges of the thermally evaporated PbI_2 layer on ZnO/FTO/glass substrate. a, b, The top view (**a**) and cross-sectional (**b**) SEM images of the thermally evaporated PbI_2 layer grown on ZnO. The processing parameters for the thermal evaporating condition are identical to the ones used for the layers shown in Fig. 1 where PbI_2 is grown on PCBM.

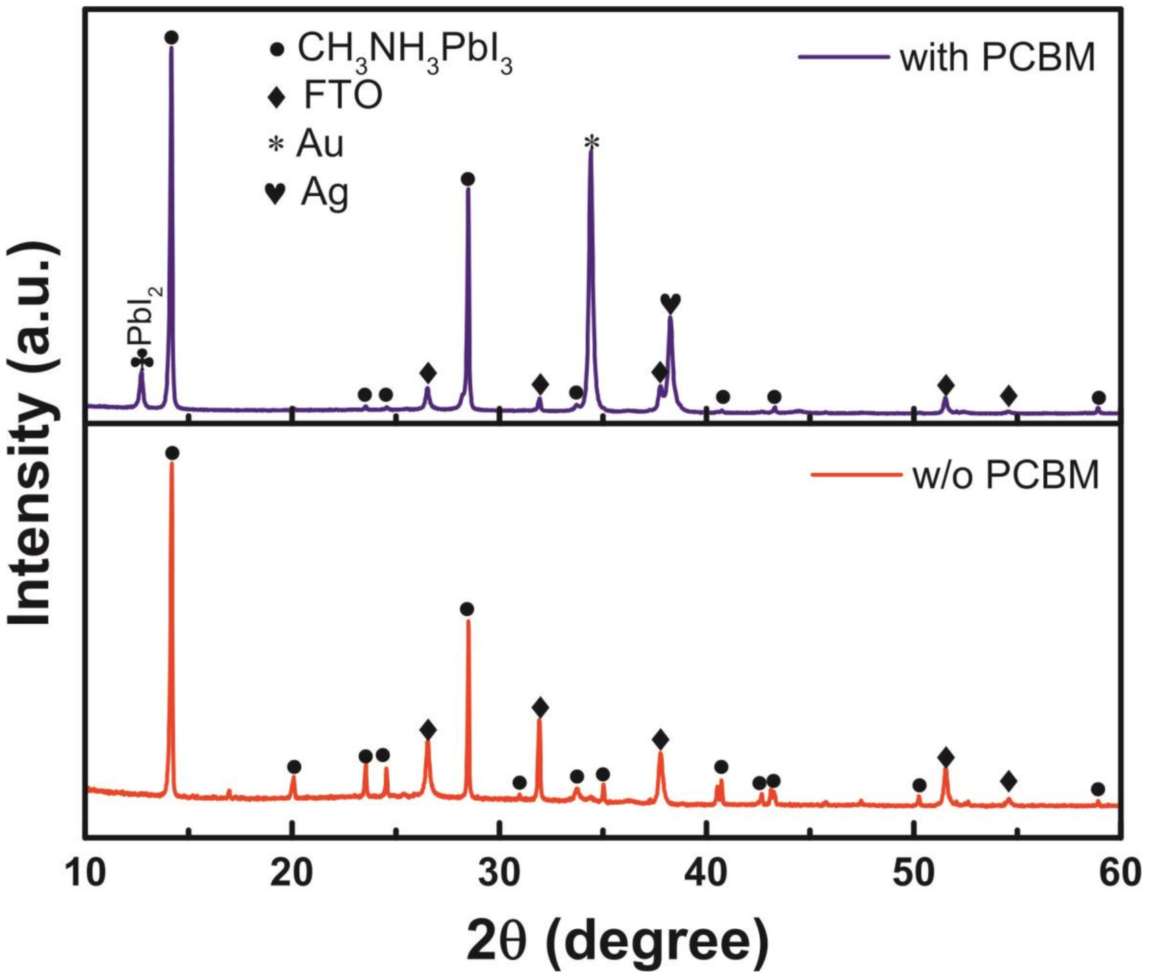

Supplementary Figure 2 | X-ray diffraction patterns of perovskite layer grown with and without PCBM under identical condition. The PbI_2 peak is observed in sample with PCBM layer, while it is not detectable in sample without PCBM. In later case, the porous PbI_2 layer consisting of high aspect ratio nanoplates facilitate the infiltration of CH_3NH_3I into PbI_2, thus lead to complete conversion of PbI_2 into perovskite. The Au and Ag peaks are observed in the sample with PCBM since a complete solar cell was measured.

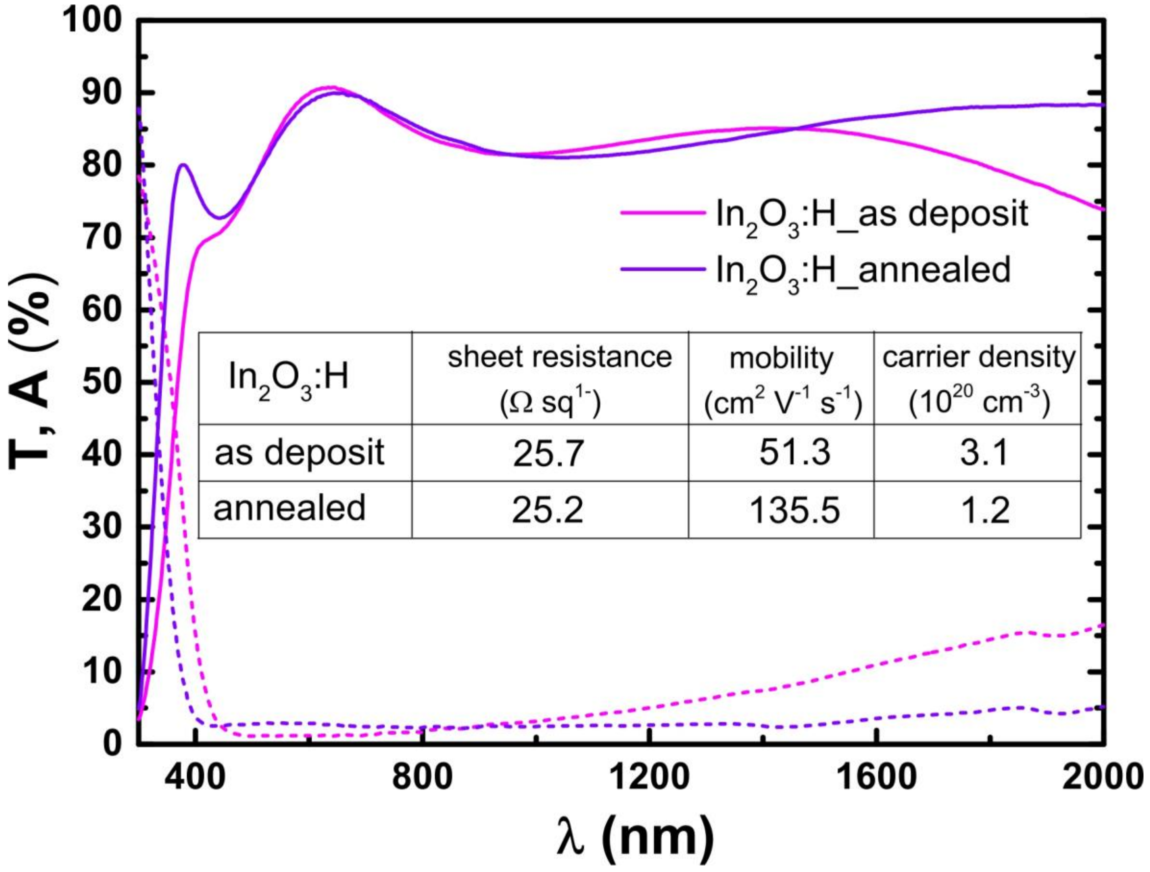

Supplementary Figure 3 | The effect of post-annealing on electrical and optical properties of In_2O_3:H film on glass. The deposition conditions for In_2O_3:H with and without annealing were identical as they were deposited in the same run. The thickness of In_2O_3:H layer is around 149 nm determined by profilometer. The post-annealing treatment is done at 200 **°C** for 2 hour in vacuum. After thermal annealing, electron mobility increased considerably, while the electron density decreased drastically, resulting in marginal change in conductivity. The solid and dashes lines indicate transmission and absorption, respectively.

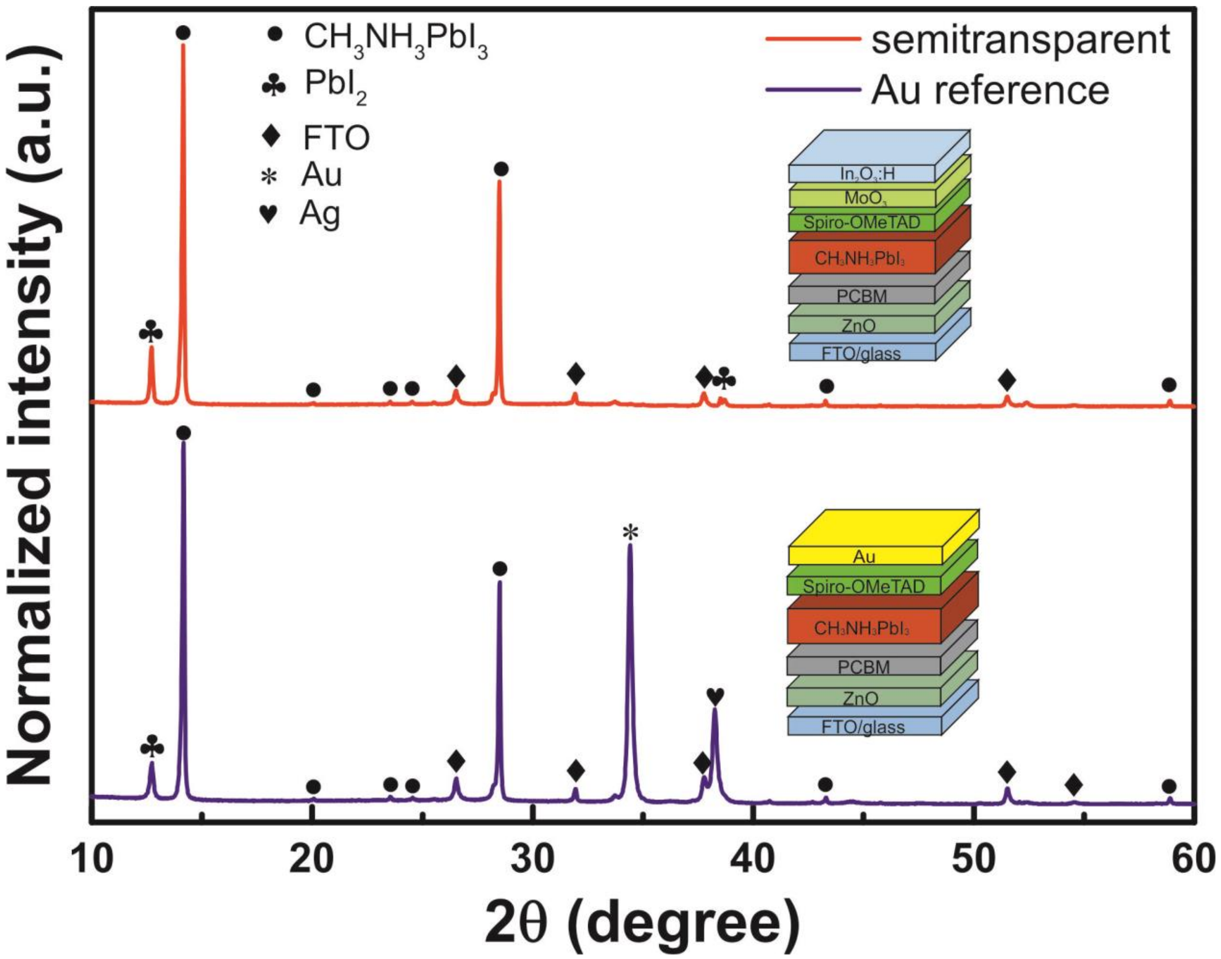

Supplementary Figure 4 | X-ray diffraction patterns of opaque and semi-transparent planar perovskite solar cells. In both devices, residual PbI_2 is observed. In the semi-transparent cell, the peaks of In_2O_3:H are absent, indicating an amorphous phase of In_2O_3:H prepared by room-temperature RF-magnetron sputtering.

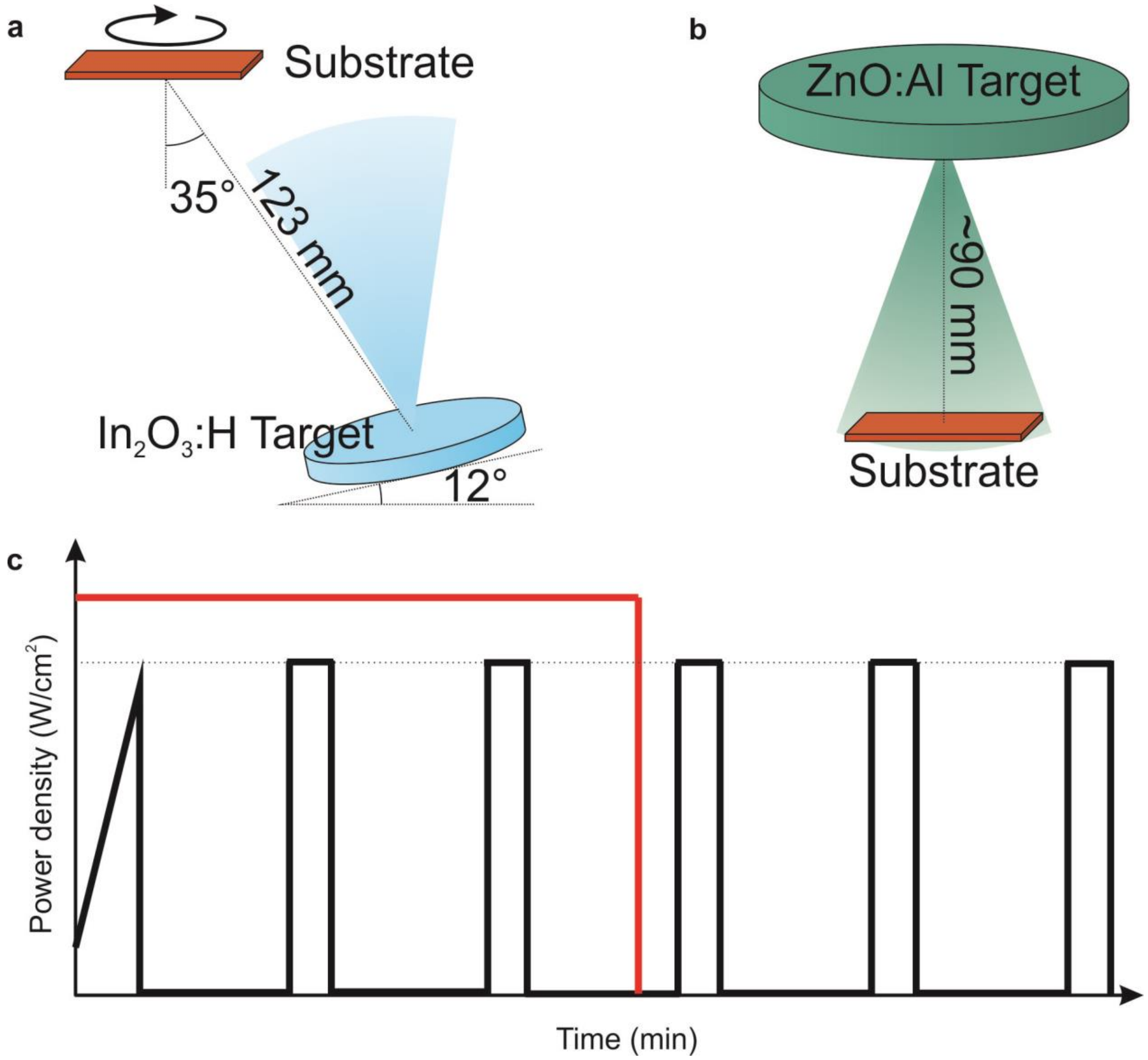

Supplementary Figure 5 | Comparison of the schematic RF-magnetron sputtering geometry and deposition procedures for In_2O_3:H and ZnO:Al. a, b, The schematic illustration of sputtering geometry for In_2O_3:H (**a**) and ZnO:Al (**b**). **c,** The schematic deposition procedures for In_2O_3:H and ZnO:Al. Although the deposition powder density and total deposition time in In_2O_3:H are larger than that of ZnO:Al, the degree of ion bombardment is more severe in case of ZnO:Al for the following reasons: (1) The target is directly facing the substrate during ZnO:Al deposition; while the target is off-centered in In_2O_3:H deposition, therefore the majority of the ions did not reach the substrate. (2) The substrate-to-target distance in ZnO:Al is shorter than that in In_2O_3:H. (3) The substrate is static during ZnO:Al deposition, however, the substrate is constant rotating during In_2O_3:H deposition, which could minimize the temperature effect. We observe that yellow spots would appear if the perovskite is under >5 min continuous ZnO:Al deposition, indicating a decomposition of perovskite.

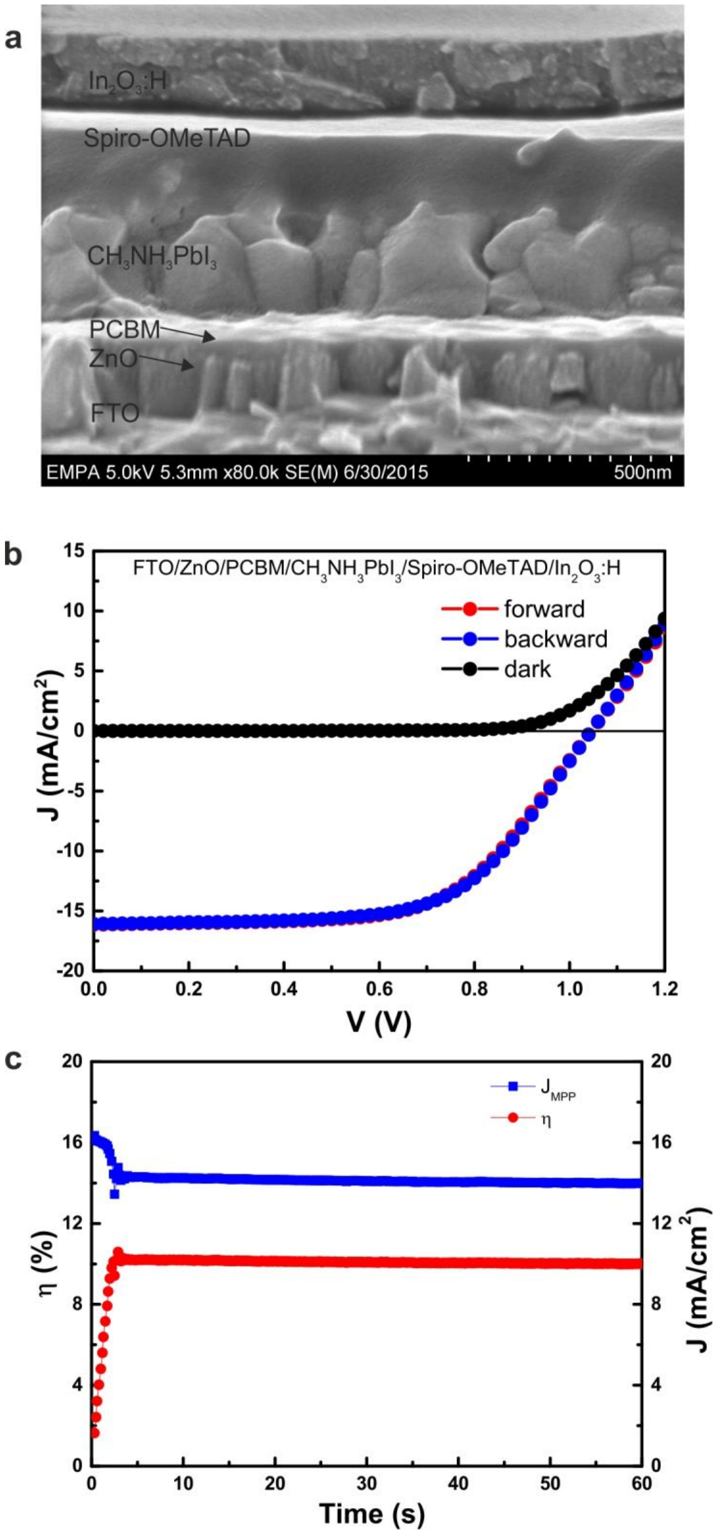

Supplementary Figure 6 | The microstructure and photovoltaic performance of semi-transparent planar perovskite solar cell without MoO_3 buffer layer. a, The cross-sectional SEM image of the complete semi-transparent device. **b, c,** The *J-V* curve (**b**) the steady-state performance (**c**) of the semi-transparent planar perovskite without MoO_3.The perovskite is grown from 140 nm PbI_2 and 45 mg/mL CH_3NH_3I in isopropanol solution.

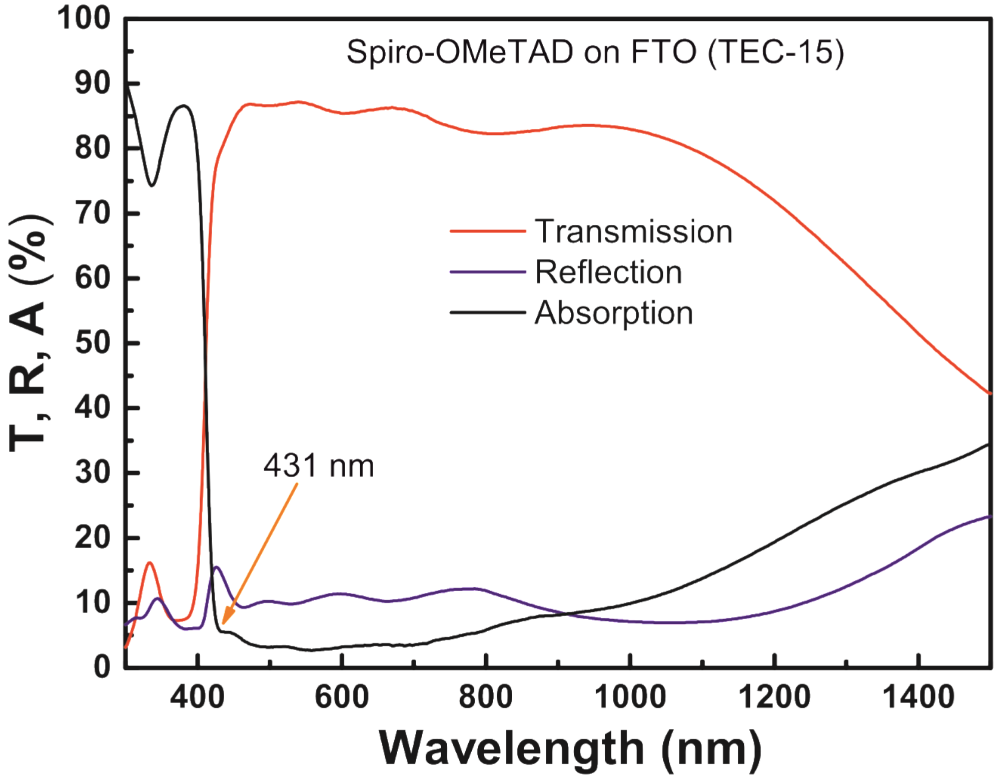

Supplementary Figure 7 | The transmission, reflection and absorption of Spiro-OMeTAD on FTO/glass (TEC-15). The deposition parameters and composition (doping concentration) of the Spiro-OMeTAD is identical to the one used in perovskite device fabrication, i.e., 30μL solution is spin coated on FTO at 2000 rpm for 40 s. There abrupt increase in absorption below 430 nm is attributed to the strong parasitic absorption from Spiro-OMeTAD.

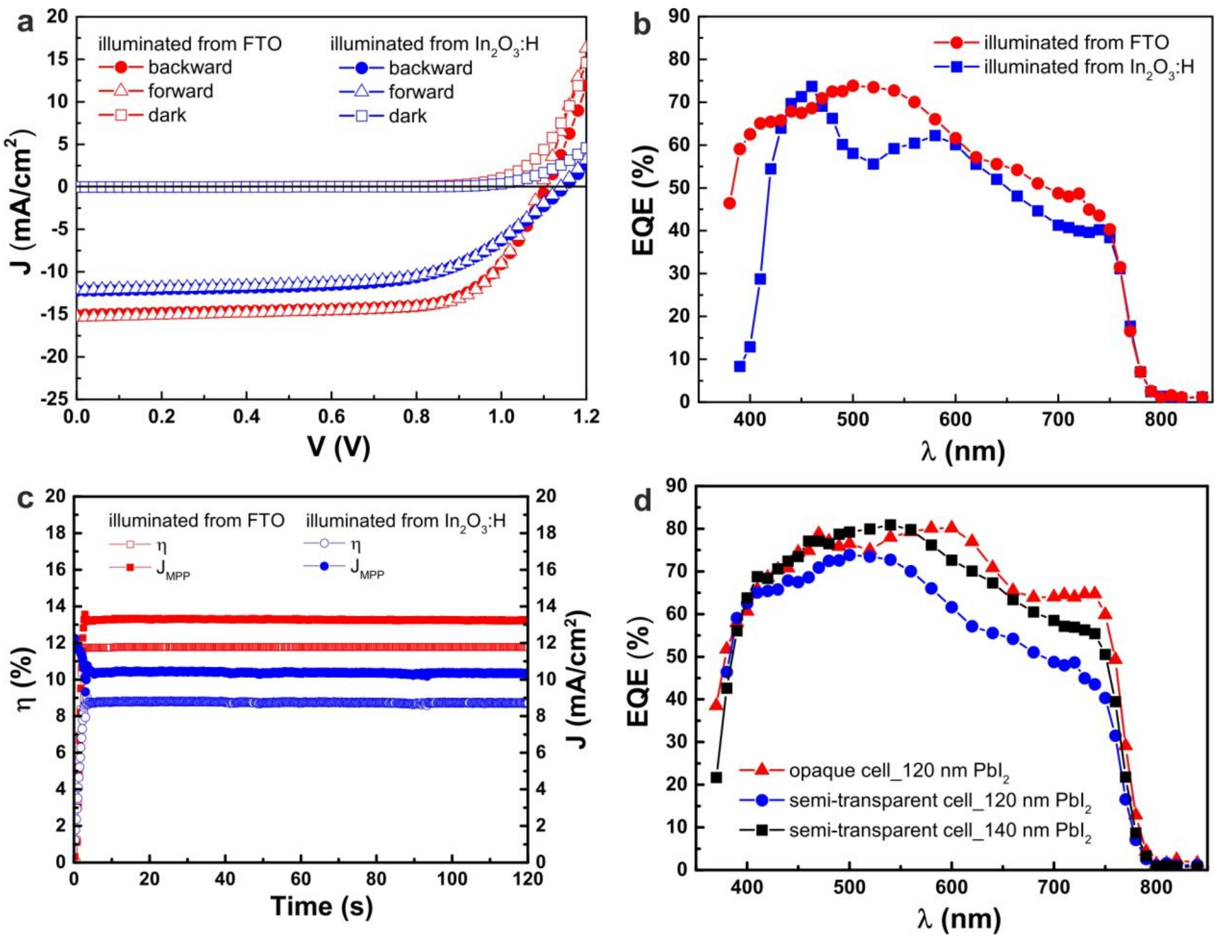

Supplementary Figure 8 | The photovoltaic performance of semitransparent planar perovskite solar cell. a, b, c, The J-V curve (**a**), EQE spectra (**b**), and steady-state performance (**c**) of semitransparent planar perovskite solar cell illuminated from both front (FTO) and rear (In_2O_3:H) side of the device. The perovskite layer is grown from 120 nm PbI_2 and 40 mg/mL CH_3NH_3I solution in isopropanol. **d**, the comparison of EQE spectra of opaque and semi-transparent devices with perovskite grown from different PbI_2 thickness.

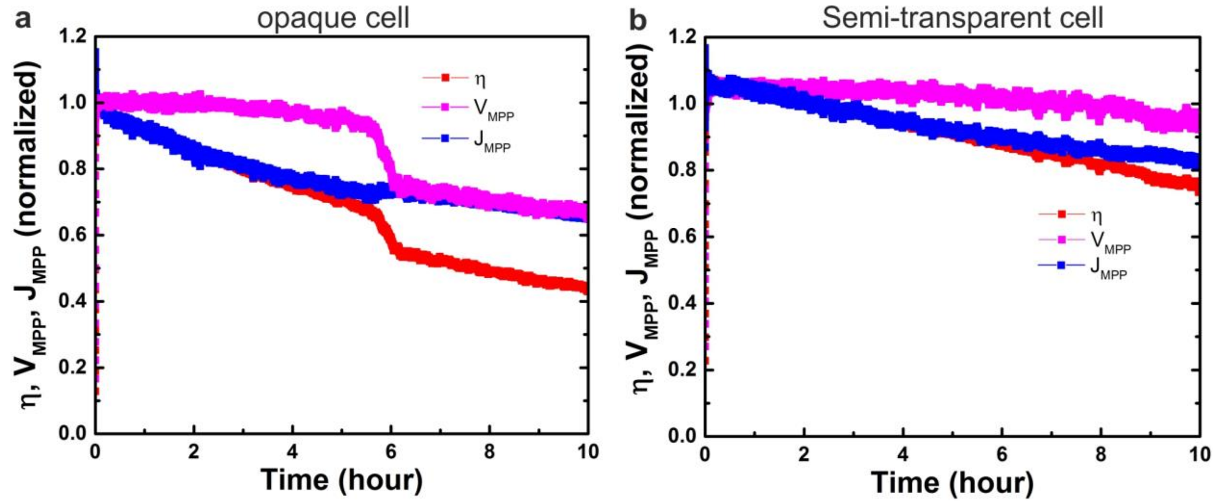

Supplementary Figure 9 | Air stability of opaque and semi-transparent perovskite solar cells. Normalized power conversion efficiency (η), V_{mpp}, J_{mpp} of **a**, opaque perovskite solar cell, and **b**, semitransparent perovskite solar cell. The cells were measured under continuous AM1.5G simulated solar spectrum (100 mW cm^{-2}) at the maximum power point (MPP) in ambient air without encapsulation and active cooling. The relative humidity during the measurement is around 40%, and no UV filter is applied. We note that all devices had initial power conversion efficiency of at least 10%.

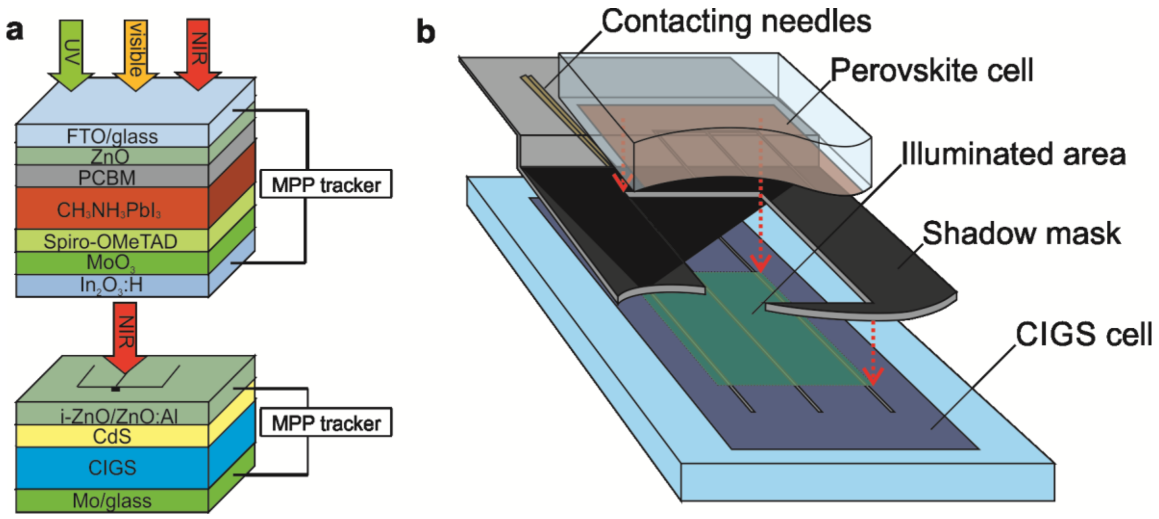

Supplementary Figure 10 | Schematic drawing of 4-terminal tandem measurements. a, Schematic 4-terminal tandem solar cell. **b,** Schematic illustration of efficiency measurement of CIGS in tandem configuration.

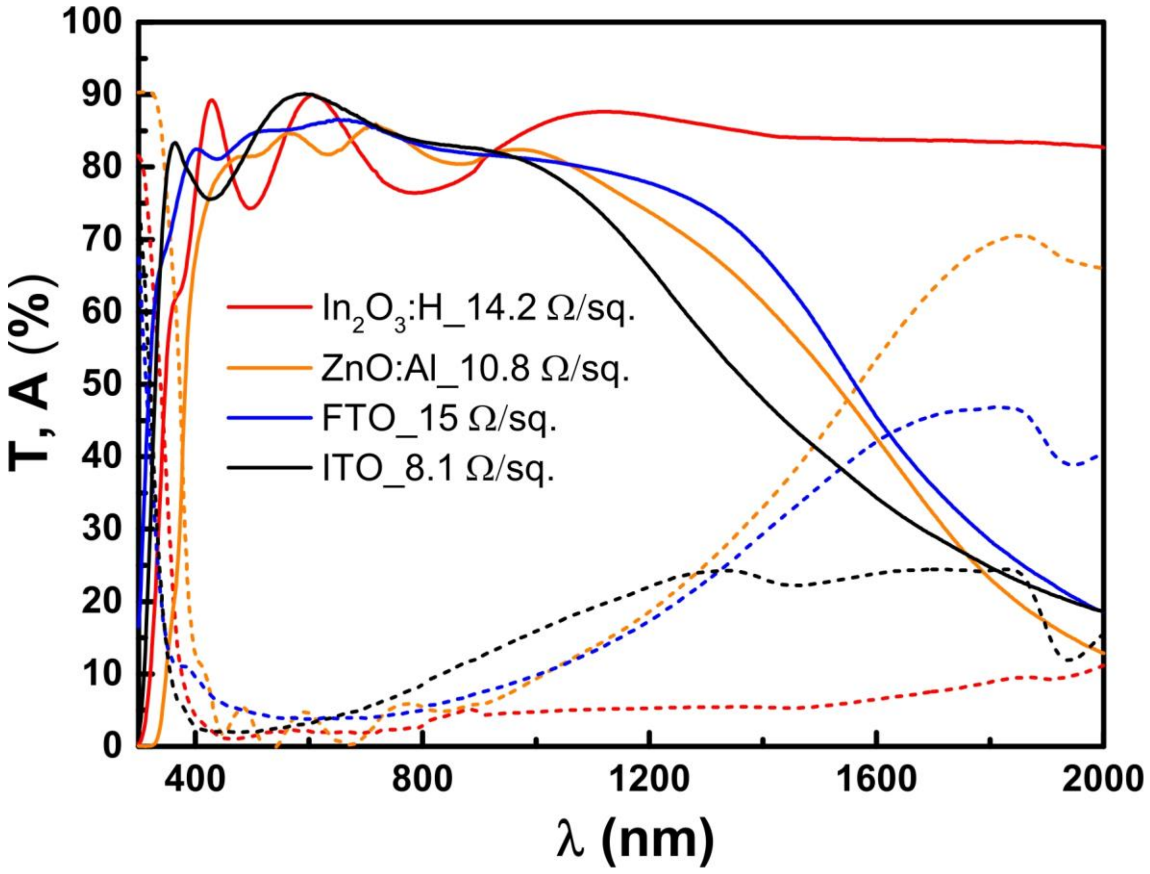

Supplementary Figure 11 | Transmission and absorption spectra of transparent conducting oxides. The In_2O_3:H and ZnO:Al is deposited in our lab, while the FTO and ITO are bought from Pilkinton and Kaivo. The In_2O_3:H was annealed at 200 **°C** for 2 hour in vacuum after deposition to increase the carrier mobility.

Solar cell	Illumination side	Scan direction	V_{OC} (mV)	J_{SC} (mA/cm^2)	*FF* (%)	η (%)	Area (cm^2)
Opaque (with PCBM)	FTO	forward	1101	17.6	74.9	14.5	0.15
		backward	1103	17.5	74.6	14.4	
Opaque (w/o PCBM)	FTO	forward	0.8521	16.2	6.7	0.92	0.15
		backward	0.9173	17.1	54.1	8.5	
Semitransparent	FTO	forward	1103	17.4	72.5	13.9	0.517
		backward	1104	17.3	73.6	14.1	
	In_2O_3:H	forward	1103	12.2	70.6	9.5	
		backward	1105	12.2	70.6	9.5	

Note: The perovskite layers of opaque devices were grown from 120 nm PbI_2 precursor layer, while 140 nm PbI_2 is used for the semi-transparent cell. No anti-reflection coating layer is applied to the semi-transparent cell.

Supplementary Table 1. Photovoltaic parameters for planar perovskite solar cells with opaque Au contact (with and without PCBM), and transparent MoO_3/In_2O_3:H/Ni-Al contact.
